# Bisphenol A and Infant Neonatal Neurobehavior

**DOI:** 10.1289/ehp.1104429

**Published:** 2012-03-01

**Authors:** Lois Haighton, Jeffrey W. Card, Barry Lynch, Ashley Roberts

**Affiliations:** Intertek Cantox, Mississauga, Ontario, Canada, E-mail: jcard@cantox.com

We read with interest the article “Case Report: High Prenatal Bisphenol A Exposure and Infant Neonatal Neurobehavior” by Sathyanarayana et al. (2011). In their article, the authors proposed a potential association between a single, exceedingly high concentration of urinary bisphenol A (BPA) measured at the beginning of the third trimester of pregnancy in a single case mother and a single abnormal neurological assessment conducted 4 months later in a single and apparently otherwise healthy infant at approximately 1 month of age. We have several comments and concerns about this article and its conclusions.

First, the urinary BPA results for the mother at the 16-week gestation test and just after birth were not abnormally elevated; the only elevated concentration was at the 26-week gestation test. Sathyanarayana et al. (2011) reported that the elevated BPA level was the highest of any reported in the peer-reviewed literature and that the bioanalytical laboratory repeated the analysis to confirm the result. The actual values detected in the repeated analysis were not provided in the article, precluding readers from independently concluding that the result was likely not spurious.

Second, Sathyanarayana et al. (2011) stated that at 26 weeks of gestation the majority of the BPA in the urine sample was conjugated, indicating that it had been metabolized and thus did not reflect external contamination. It should be noted that glucuronidated BPA is not biologically active. The authors did not propose a potential mechanism by which conjugated BPA may exert neurological effects.

Third, although the neurological assessment conducted on the infant at approximately 1 month of age was considered abnormal, neurological assessments at 14 hr after birth and annually at 1–5 years of age were within normal limits, suggesting a spurious event. Also, it is unclear whether “normal limits” refers to results at 1–5 years of age for children in the Health Outcomes and Measures of the Environment (HOME) Study from which this case study was generated (and with which the 1-month assessment results were compared) or to some other data set. If it is the latter, the authors would appear to have compared the child’s assessment results with two different data sets and drawn different conclusions from them without indicating whether (and how) the data sets differ.

The preceding items and other statements in the article raise doubt about the plausibility of a link between the single high urinary BPA measurement and the single abnormal neurological assessment. For example, Sathyanarayana et al. (2011) did not know how long the child exhibited symptoms after the abnormal assessment was conducted. The authors did not indicate that any follow-up tests were performed to detect ailments that may have been the cause of the abnormal findings; they stated only that there was “no obvious etiology.” The authors reported that they referred the mother to her primary physician, but the only information they provided regarding the results of the follow-up or when it occurred was that

[The] abnormal findings were not noted by any other medical assessments performed by health care providers, including the primary medical doctor for the infant. (Sathyanarayana et al. 2011)

Most strikingly, the authors stated that

Other infants within the HOME Study had abnormal neurologic examinations, but some of these mothers did not have elevated prenatal urinary BPA concentrations. These cases may have resulted from other etiologies of abnormal neurobehavior that have not yet been explored.

We argue that the case infant also may have had such etiologies that were not explored. Thus, it is unclear to us why Sathyanarayana et al. (2011) chose to conduct and report a case study on this single infant, other than the fact that the mother had an unusually high urinary BPA concentration at a single time point.

In conclusion, we feel that it is highly likely that the elevated third-trimester urinary BPA concentration had absolutely nothing to do with the single abnormal neurological assessment in the case infant. We do not consider this study to be “hypothesis generating” but rather to simply fan the flames of a topic that has received substantial media attention, much of which is overblown and not supported scientifically. We recommend that the authors consider reviewing the Bradford Hill criteria for establishing causality before suggesting and publishing possible cause-and-effect relationships based on a single case study.
